# Characterization of the Nencki Affective Picture System by discrete emotional categories (NAPS BE)

**DOI:** 10.3758/s13428-015-0620-1

**Published:** 2015-07-24

**Authors:** Monika Riegel, Łukasz Żurawski, Małgorzata Wierzba, Abnoss Moslehi, Łukasz Klocek, Marko Horvat, Anna Grabowska, Jarosław Michałowski, Katarzyna Jednoróg, Artur Marchewka

**Affiliations:** Laboratory of Brain Imaging, Neurobiology Centre, Nencki Institute of Experimental Biology, 3, Pasteur Street, 02-093 Warsaw, Poland; Laboratory of Psychophysiology, Department of Neurophysiology, Nencki Institute of Experimental Biology, Warsaw, Poland; University of Social Sciences and Humanities, Warsaw, Poland; Faculty of Psychology, University of Warsaw, Warsaw, Poland; Department of Computer Science and Information Technology, University of Applied Sciences, Zagreb, Croatia

**Keywords:** Basic emotion, Visual stimuli, Affective ratings, Valence, Arousal, Happiness, Fear, Sadness, Surprise, Anger, Disgust, Nencki Affective Picture System

## Abstract

The Nencki Affective Picture System (NAPS; Marchewka, Żurawski, Jednoróg, & Grabowska, *Behavior Research Methods*, 2014) is a standardized set of 1,356 realistic, high-quality photographs divided into five categories (people, faces, animals, objects, and landscapes). NAPS has been primarily standardized along the affective dimensions of valence, arousal, and approach–avoidance, yet the characteristics of discrete emotions expressed by the images have not been investigated thus far. The aim of the present study was to collect normative ratings according to categorical models of emotions. A subset of 510 images from the original NAPS set was selected in order to proportionally cover the whole dimensional affective space. Among these, using three available classification methods, we identified images eliciting distinguishable discrete emotions. We introduce the basic-emotion normative ratings for the Nencki Affective Picture System (NAPS BE), which will allow researchers to control and manipulate stimulus properties specifically for their experimental questions of interest. The NAPS BE system is freely accessible to the scientific community for noncommercial use as supplementary materials to this article.

**Electronic supplementary material:**

The online version of this article (doi:10.3758/s13428-015-0620-1) contains supplementary material, which is available to authorized users.

Given that there is no single gold-standard method for the measurement of emotion, researchers are often faced with a need to select appropriate and controlled stimuli for inducing specific emotional states (Gerrards-Hesse, Spies, & Hesse, 1994; Mauss & Robinson, 2009; Scherer, 2005). The Nencki Affective Picture System (NAPS; 2014) is a set of 1,356 photographs divided into five content categories (people, faces, animals, objects, and landscapes). All of the photographs have been standardized on the basis of dimensional theories of emotions, according to which several fundamental dimensions can characterize each affective experience. In the case of the NAPS, these dimensions are valence (ranging from *highly negative* to *highly positive*), arousal (ranging from *relaxed*/*unaroused* to *excited*/*aroused*), and approach–avoidance (ranging from a *tendency to avoid* to a *tendency to approach* a stimulus) (Osgood, Suci, & Tannenbaum, 1957; Russell, 2003). Although the identity and number of dimensions have been debated (Fontaine, Scherer, Roesch, & Ellsworth, 2007; Stanley & Meyer, 2009), this approach has been successfully used in many studies and has provided much insight into affective experience (Bayer, Sommer, & Schacht, 2010; Briesemeister, Kuchinke, & Jacobs, 2014; Colibazzi et al., 2010; Kassam, Markey, Cherkassky, Loewenstein, & Just, 2013; Viinikainen et al., 2010).

As a different way to conceptualize human emotions, they can be categorized in terms of discrete emotional states (Darwin, 1872; Ekman, 1992; Panksepp, 1992), and each emotion has unique experiential, physiological, and behavioral correlates. As was stated by Ekman (1992) in his theory of basic emotions, “a number of separate emotions . . . differ one from another in important ways” (p. 170). In line with this theoretical framework, researchers argue that one- or two-dimensional representations fail to capture important aspects of the emotional experience and do not reflect critical differences between certain emotions (Remmington, Fabrigar, & Visser, 2000). Instead, at least five different discrete emotion categories are proposed to reflect facial or vocal expression, namely: happiness, sadness, anger, fear, and disgust. By using the term “basic emotions,” Ekman (1992) wanted to indicate that “evolution played an important role in shaping both the unique and the common features which these emotions display as well as their current function” (p. 170). They are supposed to originate from biological markers, regardless of any cultural differences (Ekman, 1993). This categorical model of emotions has also provided numerous empirical insights (Briesemeister, Kuchinke, & Jacobs, 2011a; Mikels et al., 2005; Silva, Montant, Ponz, & Ziegler, 2012; Stevenson, Mikels, & James, 2007; Tettamanti et al., 2012; Vytal & Hamann, 2010).

A longstanding dispute concerning whether emotions are better conceptualized in terms of discrete categories or underlying dimensions has gained new insight from different methods in the domain of neuroimaging (see Briesemeister, Kuchinke, Jacobs, & Braun, 2015; Fusar-Poli et al., 2009; Kassam et al., 2013; Lindquist, Wager, Kober, Bliss-Moreau, & Barrett, 2012). Although some studies have identified consistent neural correlates that are associated with basic emotions and affective dimensions, the studies have ruled out simple one-to-one mappings between emotions and brain regions. This points to the need for more complex, network-based representations of emotions (Hamann, 2012; Saarimaki et al., 2015). Given that both discrete emotion and dimensional theories are greatly overlapping in their explanatory values (Reisenzein, 1994), further experimental investigations are needed using combined approaches (Briesemeister, Kuchinke, & Jacobs, 2014; Briesemeister et al., 2015; Eerola & Vuoskoski, 2011; Hinojosa et al., 2015). Therefore, providing appropriate pictorial stimuli combining both perspectives will be of great usefulness.

To meet this need, many of the existing datasets of standardized stimuli in various modalities that were originally assessed in line with the dimensional approach have now received complementary ratings on the expressed emotion categories (Briesemeister, Kuchinke, & Jacobs, 2011b; Mikels et al., 2005; Stevenson et al., 2007; Stevenson & James, 2008). Due to this contribution, it has become possible to investigate various topics in affective neuroscience, such as temporal and spatial neural dynamics in the perception of basic emotions from complex scenes (Costa et al., 2014), or the neural correlates of different attentional strategies during affective picture processing (Schienle, Wabnegger, Schoengassner, & Scharmüller, 2014). With such stimuli, different theories of emotion processing and their applicability to affective processing studies have also been examined (Briesemeister et al., 2015). Moreover, basic emotion ratings have enabled researchers to select pictures in order to study the neural correlates of affective experience and therapeutic effects in different clinical populations (Delaveau et al., 2011). In this way, a combination of the dimensional approach, useful to describe a number of broad features of emotion, and the categorical approach, focused on capturing discrete emotional responses, is supplying researchers with a more complete view of affect.

The aim of the present study was to provide researchers with a list of reliable discrete emotion norms for a subset of images selected from the Nencki Affective Picture System as being characterized both with the intensities of basic emotions (happiness, anger, fear, disgust, sadness, and surprise) and the affective dimensions of valence and arousal. Additionally, the obtained ratings are going to be analyzed for the problems of the relationship between affective dimensions and basic emotions and the relations between the affective variables and the content categories. This subset hereafter is referred to as NAPS BE.

## Method

### Materials

A subset of 510 images was selected from the NAPS database in order to proportionally cover the dimensional affective space across the content categories of animals, faces, landscapes, objects, and people. The selection was driven by reports showing that in the International Affective Picture System (IAPS; Bradley & Lang, 2007), the distribution of stimuli across the valence and arousal dimensions is related to human versus inanimate picture content (Colden, Bruder, & Manstead, 2008). Specifically, pictures depicting humans were over-represented in the high arousal–positive and high arousal–negative areas of affective space, as compared to inanimate objects, which were especially frequent in the low arousal–neutral valence area. In order to avoid a similar pattern in our dataset, and to provide a variety of stimulus content for the basic emotion classification, we chose and counterbalanced pictures from each content category covering the whole affective space. Also, we aimed at limiting the number of neutral stimuli in each subset. In this way, we obtained the following numbers of images per category: 98 animals, 161 faces, 49 landscapes, 102 objects, and 100 people. The landscape category was the least numerous, since these pictures were predominantly not arousing and of neutral valence. The NAPS BE images that proportionally covered the dimensional affective space of valence and arousal across the content categories of animals, faces, landscapes, objects, and people are depicted on Fig. S1 (supplementary materials).

### Participants

A total of 124 healthy volunteers (67 females, 57 males; mean age = 22.95 years, *SD* = 3.76, range = 19 to 37) without history of any neurological illness or treatment with psychoactive drugs took part in the study. The participants were mainly Erasmus (European student exchange programme) students from various European countries recruited at the University of Warsaw and the University of Zagreb. All of them were proficient speakers of English, and the procedure was conducted in English in order to obtain more universal norms. All of the participants obtained a financial reward of 30 PLN (approximately EUR 7).

### Procedure

Participants were first asked to fill in the informed consent form and to read instructions displayed on the computer screen (see the Appendix), then they familiarized themselves with their task in a short training session with exemplary stimuli. All of the participants were informed that in case of feeling any discomfort due to the content of the pictures, they should report it immediately to stop the experimental session. English was the language of the instructions, rating scales, and communication with the participants. During the experiment, they individually rated images through a platform available on a local server, with an average distance of 60 cm from the computer screen.

Each participant was exposed to a series of 170 images chosen pseudorandomly from all of the categories and presented consecutively under the following constraints: No more than two pictures from each affective valence category (positive, neutral, and negative) and no more than three pictures from each content category appeared consecutively. In order to avoid serial position (primacy and recency) effects, each subset of 170 pictures was divided into three parts; these parts were positioned in three possible ways and were counterbalanced across the participants.

Single images were presented in a full-screen view for 2 s. Each presentation was followed by an exposure of the rating scales (for the assessment of the basic emotions and affective dimensions) on a new screen with a smaller picture presented in the upper part of the screen. The task of the participants was to evaluate each picture on the eight scales described below. Completing the task with no time constraints took approximately 45–60 min. The local Research Ethics Committee in Warsaw approved the experimental protocol of the study.

### Rating scales

**Fig. 1:**
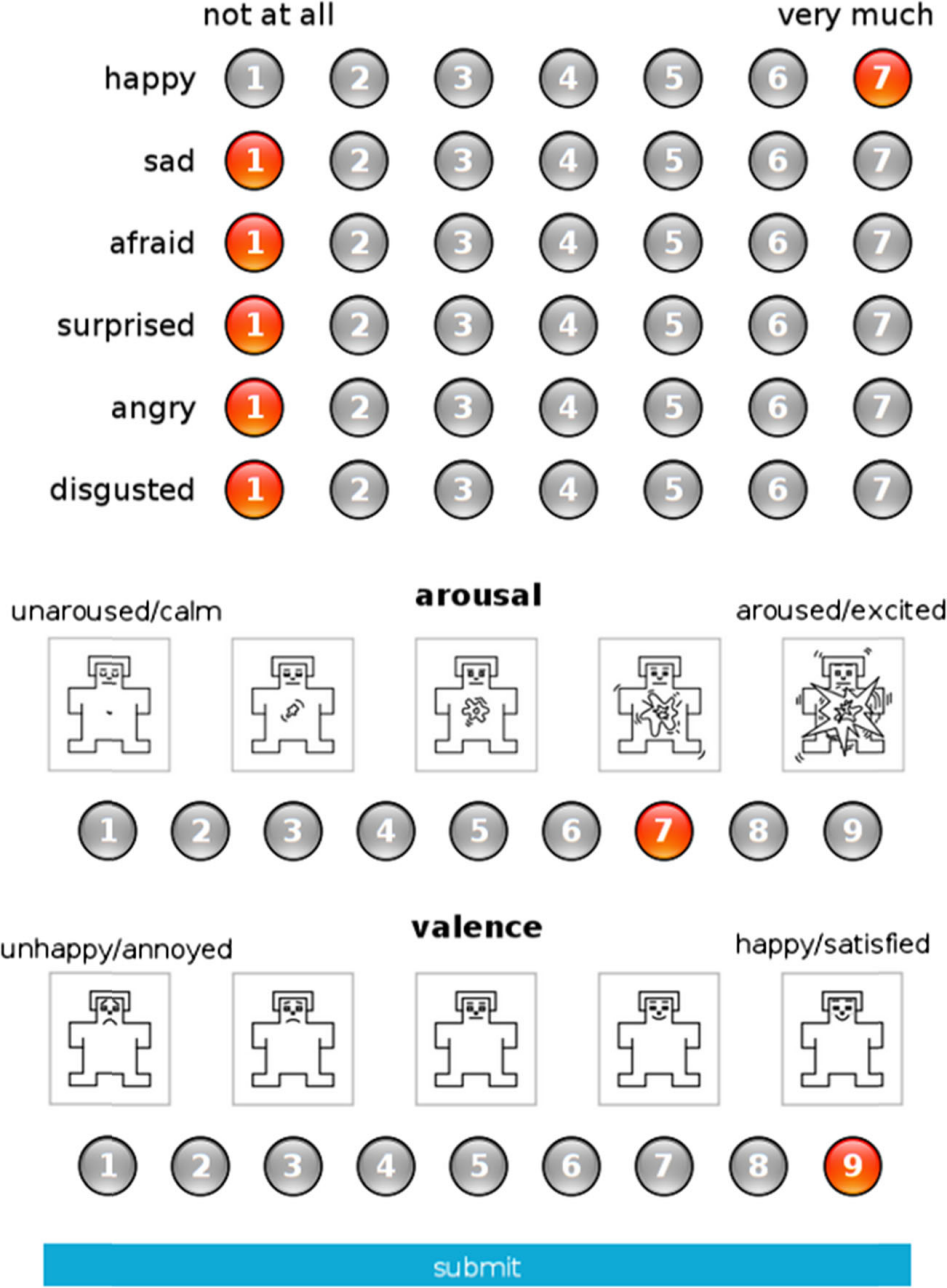
Example screen of the assessment platform for a single image, along with the discrete and dimensional scales

Analogously to some previously used procedures (Briesemeister et al., 2011b; Mikels et al., 2005; Stevenson et al., 2007), participants were asked to use six independent 7-point Likert scales to indicate the intensity of the feelings of happiness, anger, fear, sadness, disgust, and surprise (with 1 indicating *not at all* and 7 indicating *very much*) elicited by each presented image, as is presented in Fig. 1. This procedure allowed the participants to indicate multiple labels for a given image. Although surprise has been considered by some researchers to be a neutral cognitive state (Ortony & Turner, 1990) rather than an emotion, and therefore does not appear in certain classifications of the basic emotions (Ekman, 1999; Izard, 2009), it was also included in the ratings.

Additionally, the pictures were rated on two affective dimensions using the Self-Assessment Manikin (Lang, 1980), as is also presented in Fig. 1. The scale of emotional valence was used to estimate the extent of the positive or negative reaction evoked by a given picture, ranging from 1 to 9 (1 for *very negative emotions* and 9 for *very positive emotions*). On the scale of arousal, participants estimated to what extent a particular picture made them feel unaroused or aroused, ranging from 1 to 9 (1 for *unaroused*/*relaxed* and 9 for *very much aroused*—e.g., jittery or excited). Although the ratings of these two affective dimensions were originally included in NAPS, they were previously obtained with the use of continuous bipolar semantic sliding scales (SLIDER) by moving a bar over a horizontal scale. The original NAPS ratings showed a more linear association between the valence and arousal dimensions, as compared to the “boomerang-shaped” relationship found, for instance, in our sample and in the IAPS database (Lang, Bradley, & Cuthbert, 2008).

### Data analysis

The data analysis is arranged into three sections. First, we investigated whether the obtained ratings were consistent across the individuals taking part in the experiment and what was the upper limit of the correlations. Therefore, we addressed the issue of the consistency of the collected ratings, applying split-half reliability estimation. Second, we described the distributions of the norms in order to provide researchers with useful characteristics of the dataset. Although the ratings of each basic emotion were given for each picture (provided in the supplementary materials, Table S2), we were also interested in searching for the pictures expressing specifically one basic emotion much more than the others. Thus, we used several methods for classifying pictures to particular basic emotions, which we consider to be useful for more precise experimental manipulations of the NAPS BE stimuli. The last section is devoted to further analyses of the patterns observed in the obtained ratings and addresses the potential doubts of researchers. In order to give a rationale for combining the theoretical frameworks of affective dimensions and basic emotions instead of choosing only one, we investigated the relationship between these approaches. Our research question was whether the information collected by emotional categories represented the same emotional information described by the dimensional ratings. To answer this question, regression analyses were performed, using the categorical data for each picture to predict the dimensional data, and vice versa. Finally, we aimed at showing researchers that other stimulus parameters are important for their experiments. Our research question was whether there were any differences in the mean basic emotion intensities across the content categories of the pictures. We investigated this relation with multivariate analysis of variance (MANOVA), considering Content Category (animals, faces, landscapes, objects, and people) and classes of the pictures’ Valence (negative, neutral, and positive) as between-subjects factors, and all of the affective ratings as dependent variables. Answering these research questions should encourage future users of NAPS BE to use all of the provided norms and variables in their experiments.

## Results

### Reliability

Since the applicability of the collected affective norms in experimental studies is highly dependent on their reliability, we addressed this issue by applying split-half reliability estimation, following descriptions provided in the literature (Monnier & Syssau, 2014; Montefinese, Ambrosini, Fairfield, & Mammarella, 2014; Moors et al., 2013). The whole sample was split into halves in order to form two groups with the odd and even experiment entrance ranks. Within each group, the mean ratings of each basic emotion were calculated for each picture. Pairwise Pearson’s correlation coefficients of these means between the two groups were then calculated and adjusted using the Spearman–Brown formula. All correlations were significant (*p* < .01). The obtained reliability coefficients were high and comparable to the values obtained in other datasets of standardized stimuli (Bradley & Lang, 2007; Imbir, 2014; Monnier & Syssau, 2014; Moors et al., 2013)- namely, *r* = .97 for happiness, *r* = .98 for sadness, *r* = .93 for fear, *r* = .94 for surprise, *r* = .95 for anger, *r* = .97 for disgust, *r* = .93 for arousal, and *r* = .98 for valence.

### Ratings of the affective variables

For each picture, we obtained from 39 to 44 ratings (*M* = 41.33, *SD* = 2.06) on each scale from the 124 participants of the study. In order to further explore the present data, we divided the whole set of pictures by their valence classes into negative, neutral, and positive pictures, according to the criteria introduced in previous studies (e.g., Ferré, Guasch, Moldovan, & Sánchez-Casas, 2012; Kissler, Herbert, Peyk, & Junghofer, 2007). These criteria were based on the mean valences for negative, neutral, and positive pictures, which usually took values around 2, 5, and 7, respectively. Therefore, we classified pictures with values of valence ranging from 1 to 4 as *negative* (*M* = 3.10, *SD* = 0.58), pictures with values ranging from 4 to 6 as *neutral* (*M* = 5.02, *SD* = 0.55), and pictures with values ranging from 6 to 9 as *positive* (*M* = 6.52, *SD* = 0.39). These criteria resulted in the following proportions in the present database: 148 negative pictures (28.6 %), 203 neutral pictures (40.8 %), and 159 positive pictures (30.6 %). The following distributions of negative, neutral, and positive pictures were observed in the different content categories: animals (25.5 % negative, 43.9 % neutral, 30.6 % positive), faces (26.1 % negative, 30.4 % neutral, 43.5 % positive), landscapes (12.2 % negative, 38.8 % neutral, 49.0 % positive), objects (19.6 % negative, 69.6 % neutral, 10.8 % positive), people (55.0 % negative, 21.0 % neutral, 24.0 % positive).

**Fig. 2:**
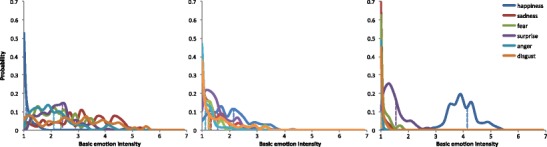
Distributions of the ratings of discrete emotion categories (happiness, sadness, fear, surprise, anger, and disgust), together with the medians of the respective distributions (dotted lines), for the negative (left), neutral (middle), and positive (right) pictures in NAPS BE

The distributions of all of the basic emotions, as collected for each picture and with pictures divided by their valence classes, are depicted in Fig. 2. We split the full range of the basic emotions (1–7 on the rating scales) into seven bins. For each bin, the number of means falling within the bin range was calculated for each basic emotion separately. Numbers obtained in this way (normalized by dividing them by the number of pictures in a particular valence class) are plotted for each valence class separately in each of the panels of Fig. 2.

The distributions of all the basic-emotion intensity ratings among negative pictures seem to be skewed, with a strong bias toward the low range of the scale. Only 31 % and 23 % of the pictures were rated above the middle value of the rating scales (=4) for sadness and disgust, respectively. All of the other basic emotion intensities were almost always rated lower. This low-intensity bias, which stands for a relative lack of pictures presenting high-intensity values of basic emotions, is strongest for happiness and surprise and weakest for sadness and disgust. All of the basic emotion intensities were rated low among the neutral pictures, with the highest median value being for happiness (Mdn = 2.15). In the positive picture group, the distribution of happiness covers the middle of the rating scale, Mdn = 4.15.

### Basic-emotion classification

The analysis above shows that the majority of images do not express just one discrete emotion, but rather are associated with several different emotional states. Therefore, from the practical point of view it might be important to select stimuli representing one particular emotion much more than any other. Such images will be very useful for further studies in which an emotional category is considered an important factor (Briesemeister et al., 2015; Chapman, Johannes, Poppenk, Moscovitch, & Anderson, 2012; Costa et al., 2014; Croucher, Calder, Ramponi, Barnard, & Murphy, 2011; Flom, Janis, Garcia, & Kirwan, 2014; Schienle et al., 2014; van Hooff, van Buuringen, El M’rabet, de Gier, & van Zalingen, 2014). Importantly, several methods of stimulus classification according to the basic emotion categories available in the literature (Briesemeister et al., 2011b; Mikels et al., 2005) can be employed, depending on the specific interest of the researcher. One of the most popular is based on the overlapping of confidence intervals (CIs; Mikels et al., 2005). Using this method, the 85 % CI was constructed around the mean intensity of each basic emotion for a given picture, and a category membership was determined according to the overlap of the CIs. A single emotion category was ascribed to a given picture if the mean of one emotion was higher than the means of all of the other emotions, and if the CI for that emotion did not overlap with the CIs for the other five emotional categories. An image was classified as *blended* if two or three means were higher than the rest and if the CIs of those means overlapped only with each other. Finally, if the CIs of more than three means overlapped, such an image was classified as *undifferentiated* (Mikels et al., 2005).

**Fig. 3:**
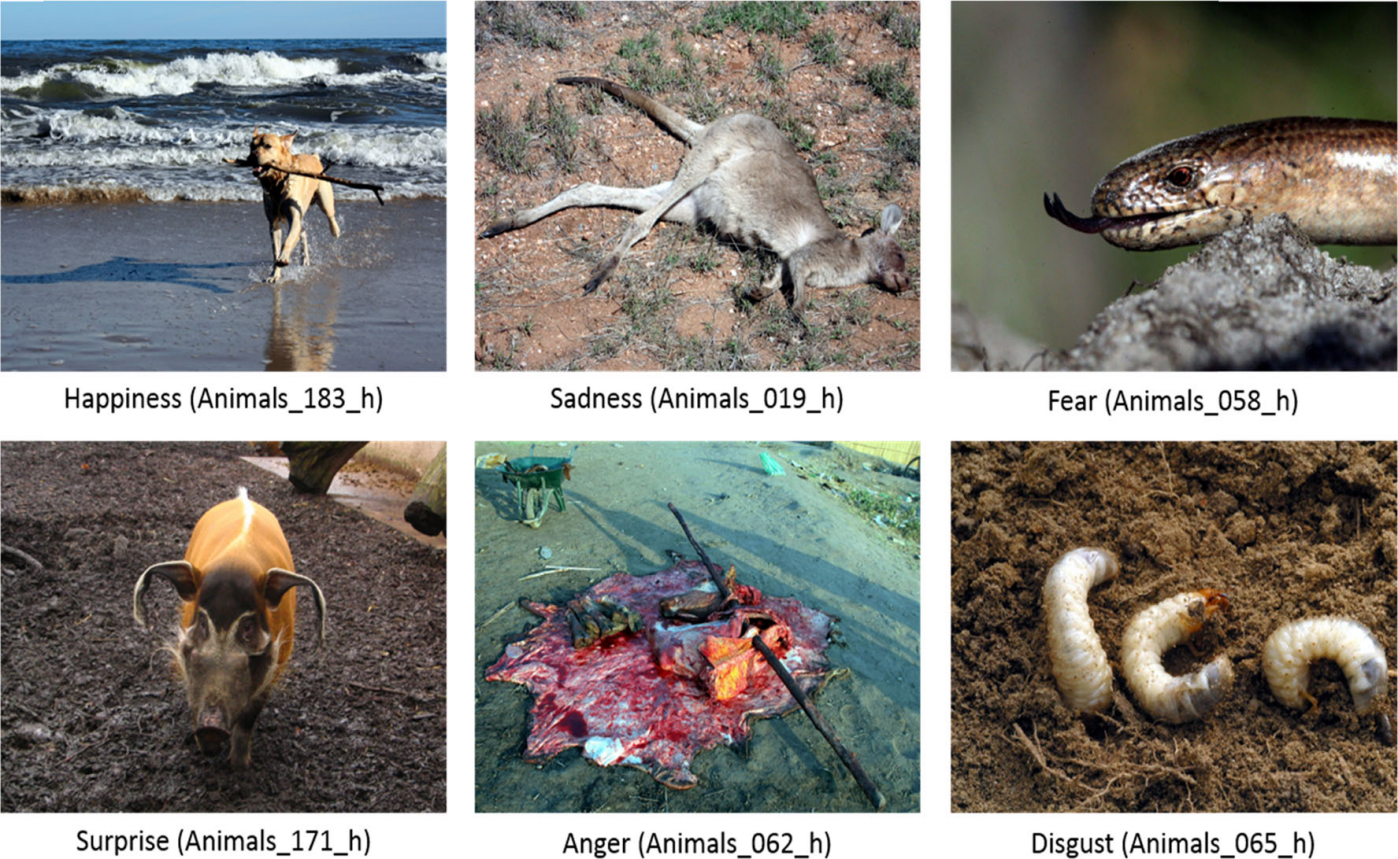
A sample of standardized images classified as representing each basic emotion within the content category of animals

The aforementioned procedure was used to find images that elicited one discrete emotion more than the others. As a result, 510 images used in the study were divided into six categories: happiness (*n* = 240), anger (*n* = 2), sadness (*n* = 62), fear (*n* = 11), disgust (*n* = 51), and surprise (*n* = 2), giving a total number of 368 pictures that were matched to specific basic emotions. The other pictures were classified as blended, including two (*n* = 21) or three (*n* = 22) emotions, or were classified as undifferentiated, eliciting similar amounts of four, five, or six emotions (*n* = 20, 25, and 54 pictures, respectively). Some example images from the animals category are presented in Fig. 3.

We computed a series of one-way analyses of variance solely on the pictures classified with the CI method (Mikels et al., 2005) as eliciting single basic emotions. For each group of pictures classified with a particular basic emotion, we compared the intensity ratings of this basic emotion in these pictures and in the pictures classified with all the other basic emotions. We obtained a significant effect of the basic-emotion classification in each case—namely, for happiness, *F*(5, 362) = 200.43, *p* < .001; sadness, *F*(5, 362) = 449.92, *p* < .001; fear, *F*(5, 362) = 147.10, *p* < .001; surprise, *F*(5, 362) = 44.19, *p* < .001; anger, *F*(5, 362) = 138.02, *p* < .001; and disgust, *F*(5, 362) = 350.14, *p* < .001.

**Fig. 4:**
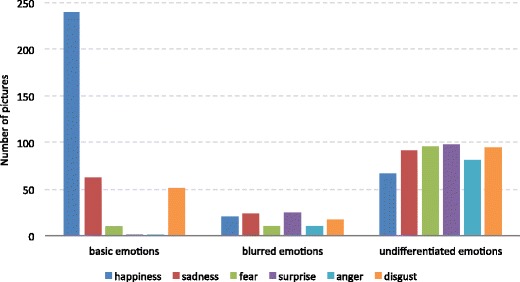
Numbers of pictures expressing each discrete emotional category, classified on the basis of confidence intervals as expressing pure, blended, and undifferentiated emotions

The frequencies of each basic emotion among the pictures classified as single, blended, and undifferentiated basic emotions are presented in Fig. 4. It is noteworthy that the three panels of this figure cannot be compared with regard to the sums of the pictures, since in the middle and right panels the same image contributed to several bars, whereas the number of pictures equals the sum of the bars in the first panel. The bars should be interpreted only in terms of the single bars informing us how often a particular emotion was represented as single, blended, or undifferentiated.

In order to provide researchers with an overview of the groups of pictures distinguished with the CI classification method, descriptive statistics for the basic emotions and affective dimensions are presented in Table 1.

**Table 1:** Descriptive statistics of all of the pictures classified by single basic emotions: Happiness, sadness, fear, surprise, anger, and disgust (*N* = 369)

	*M*	*SD*	Min	Max
Happiness	3.71	0.86	1.63	5.56
Sadness	4.04	0.77	2.28	5.49
Fear	3.30	0.33	2.71	3.70
Surprise	2.16	0.54	1.78	2.54
Anger	4.36	0.05	4.32	4.39
Disgust	3.93	0.79	1.82	5.71
Arousal	3.10	0.90	1.49	6.38
Valence	5.22	1.46	1.84	7.82

*N*, number of ratings; *M*, mean; *SD*, standard deviation; Min, minimal rating; Max, maximal rating

As was mentioned in previous studies (e.g., Mikels et al., 2005), alternative methods could be used to investigate the data. For instance, the CI method would classify images rated by one discrete emotion as having significantly higher ratings than the others, even though the intensity of this single rating was lower than those for other images that elicit blended or undifferentiated emotions. Following this, we provide a conservative classification method (Briesemeister et al., 2011b), according to which pictures were assigned to a specific discrete emotion category if the mean rating in one discrete emotion was more than one standard deviation higher than the ratings for other discrete emotions. Finally, the most liberal classification criterion was applied (Briesemeister et al., 2011b), according to which all of the pictures that received a higher mean rating in a particular discrete emotion were labeled as being related to this emotion. The results of all three classification methods are presented in Table 2.

**Table 2:** Pictures in NAPS BE representing basic emotions, as classified with confidence intervals, according to the conservative and the liberal method

		Hap	Sad	Fea	Sur	Ang	Dis	Total
Cis	Single	240	62	11	2	2	51	368
	Blended							43
	Undifferentiated							99
Conservative		153	17	0	0	0	6	176
Liberal		273	195	21	14	0	6	509

Hap, happiness; Sad, sadness; Fea, fear; Sur, surprise; Ang, anger; Dis, disgust

Since all of the methods of classification are based on means and CIs, the picture classifications of our data did not differ substantially across the three methods described above. No pictures were classified with different basic emotions according to the different methods. The only difference was the obtained numbers of pictures classified as expressing specific basic emotions. Table S2 includes the results of each classification method for each single picture.

### Relationship between basic emotions and affective dimensions

An exploration of the relationships between the basic emotions and affective dimensions showed that these variables were highly intercorrelated, as is demonstrated in Table 3.

**Table 3:** Correlations between the ratings obtained for all affective variables

	Hap	Sad	Fea	Sur	Ang	Dis	Aro	Val
Happiness	1.00^*^							
Sadness	–.67^*^	1.00^*^						
Fear	–.60^*^	.67^*^	1.00^*^					
Surprise	–.43^*^	.56^*^	.76^*^	1.00^*^				
Anger	–.62^*^	.82^*^	.66^*^	.58^*^	1.00^*^			
Disgust	–.62^*^	.52^*^	.63^*^	.71^*^	.63^*^	1.00^*^		
Arousal	–.25^*^	.64^*^	.79^*^	.74^*^	.65^*^	.61^*^	1.00^*^	
Valence	.93^*^	–.85^*^	–.75^*^	–.60^*^	–.78^*^	–.73^*^	–.53^*^	1.00^*^

^*^ *p* < .01

Additionally, regression analyses were computed using the discrete emotional category ratings in order to examine the extent to which these variables could predict the ratings of valence and arousal (Bradley & Lang, 1999). We performed four separate analyses using the six emotional category ratings to predict valence and arousal within the three valence classes distinguished in the previous sections (negative, neutral, and positive), in line with analyses reported in literature (Montefinese et al., 2014; Stevenson et al., 2007; Stevenson & James, 2008).

After removing the insignificant coefficients, we repeated the regressions; all four models turned out to fit the data, and the basic-emotion intensities explained a large percentage of the variance of valence [*F*(5, 142) = 172.41, *p* < .001, *R*^2^ = .86, for negative pictures; *F*(5, 197) = 413.99, *p* < .001, *R*^2^ = .91, for neutral pictures; and *F*(3, 155) = 182.33, *p* < .001, *R*^2^ = .78, for positive pictures] and of arousal [*F*(6, 141) = 93.47, *p* < .001, *R*^2^ = .80, for negative pictures; *F*(6, 196) = 157.52, *p* < .001, *R*^2^ = .83, for neutral pictures; and *F*(6, 152) = 59.14, *p* < .001, *R*^2^ = .70, for positive pictures].

Standardized *β* coefficients were calculated for all six emotional categories. As for negative pictures, valence was strongly related to sadness, disgust, happiness, fear, and anger, yet it was not related to surprise. Arousal, in turn, was related to fear, disgust, and sadness, but not to anger and surprise. In the case of neutral pictures, valence was most strongly related to happiness, sadness, disgust, and fear, and additionally to surprise, but not to anger. Arousal was also not related to anger, yet it was related to fear, happiness, sadness, disgust, and surprise. As far as positive pictures were concerned, valence was related to happiness, sadness, and disgust only. Arousal was related to fear, disgust, anger, and sadness (but only fear was significant).

**Fig. 5:**
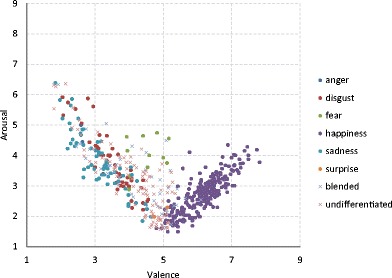
Ratings of the pictures classified on the basis of the confidence interval as basic, blended, and undifferentiated emotions in the space of the affective dimensions: valence and arousal

However, partial correlations (representing the unique influence of one predictor relative to the part of the variance of a dependent variable unexplained by the other predictors) revealed that discrete emotions contributed to valence and arousal in different ways (Ric, Alexopoulos, Muller, & Aubé, 2013) (Table 4). The ratings of affective dimensions were predicted particularly well by the level of happiness among positive pictures; by the levels of happiness, sadness, and fear among neutral pictures; and by the levels of sadness, fear, and disgust among negative pictures. The distribution of the ratings of pictures classified as eliciting particular discrete emotions on the basis of the CI criterion is presented in the affective space of valence and arousal in Fig. 5.

**Table 4:** Regressions and partial correlations of discrete emotional category ratings predicting valence and arousal, for negative, neutral, and positive words separately

	Predicting Valence			Predicting Arousal		
	*β*	*t*	Partial *r*	*β*	*t*	Partial *r*
Negative						
Happiness	.20	5.15^**^	.40	–	–	–
Sadness	–.59	–13.09^**^	–.74	.34	6.64^**^	.49
Fear	–.26	–7.21^**^	–.52	.55	12.97^**^	.74
Surprise	–	–	–	–	–	–
Anger	–.12	–3.03^**^	–.25	.09	1.97	.16
Disgust	–.33	–7.80^**^	–.55	.38	8.62^**^	.59
Neutral						
Happiness	.57	23.94^**^	.86	.32	9.49^**^	.56
Sadness	–.34	–14.36^**^	–.72	.19	5.86^**^	.39
Fear	–.21	–7.48^**^	–.47	.68	16.94^**^	.77
Surprise	.08	2.55^*^	.18	.14	3.22^**^	.22
Anger	–	–	–	–	–	–
Disgust	–.26	–10.21^**^	–.59	.20	5.65^**^	.37
Positive						
Happiness	.83	21.56^**^	.87	–	–	–
Sadness	–.13	–3.34^**^	–.26	–.08	–0.97	–.08
Fear	–	–	–	.40	5.34^**^	.40
Surprise	–	–	–	–	–	–
Anger	–	–	–	.09	1.22	.10
Disgust	–.12	–2.96^**^	–.23	–.12	–1.52	–.12

^*^ *p* < .01, ^**^ *p* < .001

The regressions calculated using the dimensional ratings to predict emotional category ratings were similar to the previous ones, also showing a lack of homogeneity in their relationships (beta weights and a statistical analysis are presented in Table S1 in the supplementary materials).

### Relations between the affective variables and the content categories

Subsequently, we performed a MANOVA including the five Content Categories (animals, faces, landscapes, objects, and people) and the three classes of Picture Valence (negative, neutral, and positive) as between-object factors, and the ratings of the six basic emotions intensities as well as the ratings of the two affective dimensions as dependent variables. Before that, we tested the assumption of the absence of multicollinearity between the dependent variables. The variance inflation factor (VIF) showed that multicollinearity might be a problem (Myers, 1990) for valence (VIF = 17.56) and happiness (VIF = 11.29). Therefore, we removed valence as a dependent variable from the analysis. Additionally, conducting collinearity diagnostics checked for interdependence of the independent variables. The obtained tolerance and VIF values were not considered problematic (tolerance > 10 and VIF < 10; Myers, 1990).

**Fig. 6:**
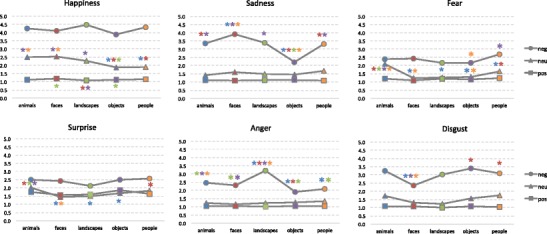
Mean intensities of all discrete emotion categories, as a function of all semantic categories and all valence classes. ^*^Significant differences between the mean intensities of particular basic emotions of content categories, marked with relevant colors: animals = blue, faces = red, landscapes = green, objects = purple, and people = orange; *p* < .05

As for the between-object effects, we found significant main effects of content category [*F*(28, 1968) = 7.99, *p* < .001, *η*_p_^2^ = .10] and valence class [*F*(14, 980) = 121.25, *p* < .001, *η*_p_^2^ = .63], as well as a significant effect of the interaction between the two [*F*(56, 3465) = 4.73, *p* < .001, *η*_p_^2^ = .07]. Further analysis of this interaction showed interesting patterns specific to each basic emotion. This interaction was further interpreted through an analysis of the simple main effects of content category performed separately for each valence class, and the results are depicted in Fig. 6. There were significant differences in the mean basic-emotion intensities among the pictures of different valence classes, depending on their content category. To start listing all of them, the ratings of happiness were lower for objects than for landscapes for both neutral and positive pictures. As far as sadness was concerned, among negative pictures the ratings were significantly higher for faces and lower for objects than for the other categories. The ratings of fear were higher than those of the other categories for people (among both negative and neutral pictures) as well as animals (among neutral pictures). As for surprise, among positive pictures these ratings were lower for animals and people than among the neutral pictures. Anger among the negative pictures was rated significantly higher for landscapes than for the other categories. Finally, disgust among the negative and neutral pictures was rated higher for objects and lower for faces than for the other content categories. All of the significant differences (*p* < .05) are marked with an asterisk in Fig. 6.

## Discussion

The present study aimed at providing categorical data that would allow NAPS to be used more generally in studies of emotion from a discrete categorical perspective, as well as providing a means of investigating the association of the dimensional and categorical approaches to the study of affect.

Concerning the relationship between affective dimensions and emotional category ratings, our findings are in line with the previous characterizations of affective stimuli, including written words (Stevenson et al., 2007), emotional faces (Olszanowski et al., 2015), and affective sounds (Stevenson & James, 2008). These results showed differences in the predictions based on categories, depending on the predicted dimension, as well as on whether the pictures were positive or negative. The regressions using the dimensional ratings to predict emotional category ratings were similar to the previous regressions, with a lack of homogeneity in the ability of the categorical ratings to predict the dimensional ratings. In other words, emotional categories cannot be extrapolated from the affective dimensions; conversely, dimensional information cannot be extrapolated from the emotional categories. The heterogeneous relationships between each emotional category and the different affective dimensions of the stimuli confirms the importance of using categorical data both independently and as a supplement to dimensional data (Stevenson et al., 2007). From a practical point of view, using both dimensional and discrete emotion classifications, the researcher could design a more ecologically valid paradigm by utilizing, for instance, negative pictures that were not biased toward any particular discrete emotion, or by using pictures evoking only a particular discrete emotion (Stevenson & James, 2008).

What can be considered a particularity of the NAPS BE dataset is the fact that sadness is related not only to low arousal. As has been stated in literature (Javela, Mercadillo, & Martín Ramírez, 2008), the definition of the elements and particular elicitors of one emotion becomes difficult when one considers that individuals could experience many negative emotions when being confronted with a certain unpleasant stimulus (Mikels et al., 2005), such as a visual scene (Bradley, Codispoti, Cuthbert, & Lang, 2001; Bradley, Codispoti, Sabatinelli, & Lang, 2001). Considering that the experiences of anger, fear, and sadness elicit similar electromyographic activity (Hu & Wan, 2003), it may be argued that these emotions are related to similar levels of arousal. For instance, anger and sadness could both be elicited with the occurrence of negative events, such as blaming others and loss (Abramson, Metalsky, & Alloy, 1989; Smith & Lazarus, 1993). Thus, these might be differentiated from each other only by considering how they are appraised, but not by the related arousal. On the other hand, according to the “core affect” theory (Barrett & Bliss-Moreau, 2009; Russell, 2003), the core affective feelings evoked during an emotion depend on the situation; for instance, fear can be pleasant and highly arousing (in a rollercoaster car) or unpleasant and less arousing (detecting bodily signs of an illness) (Wilson-Mendenhall, Barrett, & Barsalou, 2013). Therefore, there might be situations in which sadness is related to high arousal, or in which high-arousing sadness is closely related to other negative, high-arousing emotions.

Importantly, we chose images that were counterbalanced in terms of content categories, thanks to which we could explore the relationship of the affective variables and the content categories included in the NAPS. This examination revealed significant main effects of content category and valence class, indicating differences across all of them. Additionally, we found significant interactions of content category and valence class. Such interactions had been reported previously for verbal materials with regard to affective dimensions (Ferré et al., 2012), yet not for visual material and basic emotions. Further analysis of this interaction in our data showed that interesting patterns were visible, especially among positive pictures for happiness and among negative pictures for sadness, fear, surprise, anger, and disgust. Depending on the basic emotion of interest, there were differences in the ratings of various content categories: For instance, sadness was induced much less by objects, and disgust much less by faces, than were any of the other categories. These interactions show that content categories should be taken into account by researchers attempting to choose appropriate stimuli to induce specific basic emotions.

When compared to the previously offered datasets of affective pictures characterized by discrete emotions, NAPS BE offers larger samples of images expressing single basic emotions, as classified with the CI method (Mikels et al., 2005). For instance, IAPS contains fewer images expressing disgust and sadness (*n*s = 31 and 42, respectively; Bradley & Lang, 2007) than does NAPS BE (*n*s = 51 and 62). Another advantage of NAPS BE is that it enables researchers to control for the physical properties of the images (Marchewka, Żurawski, Jednoróg, & Grabowska, 2014). However, the greatest advantage of the presently introduced dataset is that it offers pictorial stimuli characterized from both dimensional and basic-emotion perspectives, which makes it extremely useful for experiments within a combined approach.

### Limitations and future directions

An important limitation of the present study, similarly to previous ones (Mikels et al., 2005), is that we were not able to differentiate representative numbers of stimuli that induce clear basic emotions such as surprise and anger. The small number of images expressing anger in NAPS BE is in line with the previous results (Mikels et al., 2005) and might be explained by the fact that it is difficult to elicit extreme unpleasantness, high effort, high certainty, and strong human agency with the passive and essentially effortless viewing of static images.

Another possible limitation is that NAPS BE, just like NAPS (Marchewka et al., 2014), lacks very positive pictures with high arousal (e.g., pictures with erotic content). However, the Erotic Subset for NAPS (NAPS ERO; Wierzba et al., 2015) has been prepared. Also, the images included in NAPS BE are only moderately inductive of basic emotions. First, this may result from the nature of static images. Second, it has been claimed that pictures evoking basic emotions of higher intensities probably also evoke different emotions (van Hooff et al., 2014). Therefore, perhaps only mild emotions can be evokedi by images classified by single basic emotions. These moderate intensities should be taken into account when investigating the specific effects of basic emotions through the use of NAPS BE.

Normative ratings were collected in a group of participants from various European and non-European countries using the English language, which could potentially have influenced the obtained results (Majid, 2012). For instance, using a nonnative language to evaluate emotions could potentially increase participants’ arousal, due to anxiety (Caldwell-Harris & Ayçiçeǧi-Dinn, 2009). Thus, future investigations of the basic emotions expressed by NAPS BE should exploit cross-linguistic variation to take into account possible principles operating between language and emotion.

Finally, we have not applied all of the possible methods of classifying emotional stimuli; for instance, we did not use a recently published method based on Euclidean distance (Wierzba et al., 2015).

Currently, we are working on dedicated software called the Nencki Affective Picture System Search Tool, which will allow researchers to choose stimuli according to the normative ratings within a combined theoretical framework.

### Electronic supplementary material

Below is the link to the electronic supplementary material.

Fig. S1 (DOC 20 kb)

Table S1 (DOC 22 kb)

## Appendix: Instructions for the picture ratings in English

Thank you for agreeing to participate in this study.

We are interested in people’s responses to pictures representing a wide spectrum of situations. For the next 50 min, you will be looking at color photographs on the computer screen.

Your task will be to evaluate each photograph on six 7-point scales, indicating the degree to which you feel happiness, surprise, sadness, anger, disgust, and fear while viewing the picture.

The far left of each scale represents total absence of the given emotion (1 = *I do not feel it at all*), while the far right of the scale represents the highest intensity of the emotion (7 = *I feel it very strongly*).

Also, fill in responses for the following two scales describing how you feel while viewing the picture.

For the arousal scale (1 = *I feel completely unaroused or calm*, and 9 = *I feel completely aroused or excited*).

For the valence scale (1 = *I feel completely happy or satisfied*, and 9 = *I feel completely unhappy or annoyed*).

There are no right or wrong answers; respond as honestly as you can. Before we start, I’*d* like you to read and sign the “informed consent” statement.

You may find some of the pictures rather disturbing; should you feel uncomfortable, feel free to quit the experiment at any time.
