# Supplementary material for: Characterization of the Nencki Affective Picture System by discrete emotional categories (NAPS BE)
Source: Behav Res Methods. 2015 Jul 24;48:600–12. doi: 10.3758/s13428-015-0620-1 (PMC4891391; doi:10.3758/s13428-015-0620-1)
Supplement: Supplementary file 1 — (DOC 20 kb) [file 13428_2015_620_MOESM1_ESM.doc]

Fig. S1 NAPS BE 510 images, as proportionally covering the dimensional affective space of valence and arousal, across the content categories of animals, faces, landscapes, objects, and people.
