# Supplementary material for: Characterization of the Nencki Affective Picture System by discrete emotional categories (NAPS BE)
Source: Behav Res Methods. 2015 Jul 24;48:600–12. doi: 10.3758/s13428-015-0620-1 (PMC4891391; doi:10.3758/s13428-015-0620-1)
Supplement: Supplementary file 2 — (DOC 22 kb) [file 13428_2015_620_MOESM2_ESM.doc]

|  | **arousal** | | | **valence** | | |
| --- | --- | --- | --- | --- | --- | --- |
| **Predicted basic emotions** | *β* | *t* | *partial r* | *β* | *t* | *partial r* |
|  |  |  |  |  |  |  |
| Negative |  |  |  |  |  |  |
| **Happiness** | 0.37 | 3.27** | 0.26 | 0.87 | 7.70** | 0.54 |
| **Sadness** | -0.34 | -3.47** | -0.28 | -0.98 | -10.00** | -0.64 |
| **Fear** | 0.88 | 9.71** | 0.63 | 0.13 | 1.41 | 0.12 |
| **Surprise** | 0.7 | 7.43** | 0.53 | -0.06 | -0.59 | -0.05 |
| **Anger** | -0.06 | -0.55 | -0.05 | -0.63 | -5.41** | -0.41 |
| **Disgust** | 0.51 | 4.01** | 0.32 | 0.07 | 0.56 | 0.05 |
| Neutral |  |  |  |  |  |  |
| **Happiness** | 0.38 | 11.03** | 0.62 | 0.95 | 27.78** | 0.89 |
| **Sadness** | 0.12 | 2.05 | 0.14 | -0.61 | -10.82** | -0.61 |
| **Fear** | 0.76 | 18.80** | 0.80 | -0.18 | -4.38** | -0.3 |
| **Surprise** | 0.68 | 12.83** | 0.67 | -0.06 | -1.10 | -0.08 |
| **Anger** | 0.32 | 5.57** | 0.37 | -0.47 | -8.13** | -0.5 |
| **Disgust** | 0.29 | 5.03** | 0.34 | -0.49 | -8.51** | -0.52 |
| Positive |  |  |  |  |  |  |
| **Happiness** | 0.28 | 4.94** | 0.37 | 0.65 | 11.47** | 0.68 |
| **Sadness** | 0.47 | 4.21** | 0.32 | -0.59 | -5.35** | -0.39 |
| **Fear** | 0.79 | 7.69** | 0.52 | -0.54 | -5.22** | -0.39 |
| **Surprise** | 0.32 | 2.73* | 0.21 | -0.29 | -1.92 | -0.15 |
| **Anger** | 0.44 | 3.88* | 0.30 | -0.49 | -4.30** | -0.33 |
| **Disgust** | 0.23 | 2.01 | 0.16 | -0.46 | -3.98** | -0.3 |
| * p < .01, ** p < .001 |  |  |  |  |  |  |

Table S1. Regressions and partial correlations of valence and arousal predicting discrete emotional category ratings for negative, neutral and positive words separately.
